# Editorial: Diabetes augmentation on vascular disease, volume II

**DOI:** 10.3389/fcvm.2025.1637222

**Published:** 2025-06-11

**Authors:** Catherine A. Reardon, Godfrey S. Getz

**Affiliations:** ^1^ Ben May Department, University of Chicago, Chicago, IL, United States; ^2^ Department of Pathology, University of Chicago, Chicago, IL, United States

**Keywords:** diabetes, autonomic neuropathy, triglyceride-glucose index, cardiomyopathy and coronary artery disease, obstructive sleep apnea, SAA, nitric oxide, inflammation

**Editorial on the Research Topic**
Diabetes augmentation on vascular disease, volume II

In 2021, we summarized eight reports addressing how diabetes augments cardiovascular disease, with a focus primarily on blood sugar and lipid regulation. In this editorial, we review ten more recent studies that expand upon this topic, incorporating systemic variables beyond glycemic and lipid control.

The first report, by Lou et al., investigated heart rate responses to exercise in patients with type 2 diabetes (T2DM), emphasizing autonomic nervous system dynamics. Typically, exercise induces sympathetic activation and parasympathetic withdrawal, which reverses during recovery. In a small Chinese cohort, this autonomic response was blunted both during and after exercise in type 2 diabetics compared to individuals without T2MD. While the authors adjusted for several confounders, the retrospective design limited control over factors such as diabetes duration, glycemic control, and medication use. Though correlative, the findings suggest a potential role for diabetic autonomic neuropathy. Future prospective studies, including larger and more diverse ethnic groups, are warranted.

A second retrospective study by Wang et al. examined the triglyceride-glucose (TyG) index as a predictor of preclinical heart failure with preserved ejection fraction (HFpEF) in a diabetic Chinese population without history of cardiovascular disease. Similar limitations as in the Lou et al. study applied, and the generalizability of the findings must be tested in broader populations.

In a cross-sectional study of 1,973 Chinese patients undergoing coronary angiography, Dong et al. found a positive correlation between glycemia and the presence of multivessel coronary artery disease (CAD), defined as >50% stenosis in at least two major coronary arteries. The association was particularly strong in males over 45 and smokers. For each unit increase in glycemia, the prevalence of multivessel CAD rose by 4%. Although cross-sectional in nature, the study underscores the potential value of glycemic measures as markers of CAD risk in Asian populations, warranting longitudinal validation in other ethnic groups.

Two Mendelian randomization studies in this Research Topic help address confounding factors common in observational research. The first by Liu et al. demonstrated a causal link between obstructive sleep apnea (OSA) and diabetic microvascular complications, including nephropathy and neuropathy in European patients. While OSA also appeared linked to diabetic retinopathy overall, the association was not significant when retinopathy was subclassified into background and proliferative forms—likely due to limited sample size. Importantly, reverse causality was not supported. Reduced lung function, as measured by forced vital capacity and expiratory volume, was also associated with increased risk of retinopathy and nephropathy.

The second Mendelian randomization study, by Feng et al., used GWAS data from individuals of European descent to explore the role of very low-density lipoprotein (VLDL) in diabetic cardiomyopathy and coronary artery disease (CAD). Type 2 diabetes was associated with a 13% increased risk of CAD and a 2.5% elevation in VLDL levels. Notably, each standard deviation increase in VLDL concentration correlated with a 30% greater likelihood of CAD, implicating VLDL as a potential mediator of diabetic cardiovascular risk.

Wang et al. conducted a bibliometric analysis of diabetic cardiomyopathy publications from 2012 to 2021, revealing a steady increase in research output, with over 250 annual publications by the decade's end. While mechanistic insights were not the focus, hyperglycemia was a recurring theme, alongside oxidative stress, fibrosis, apoptosis, and autophagy. Six institutions—primarily in China, the U.S., Australia, and Hong Kong—accounted for the majority of the contributions. Therapeutic targets were frequently discussed, highlighting the urgency of this underdiagnosed but lethal complication.

den Hartigh et al. provided a comprehensive review of serum amyloid A (SAA) proteins and their role in inflammation-related diseases, including metabolic diseases such as diabetes, obesity, and atherosclerosis. Of the five main SAA subtypes, SAA1 and SAA2 are liver-derived and markedly elevated during acute inflammation. Chronic inflammation elicits a more modest SAA response. SAA's poor solubility leads to its association with high-density lipoprotein. The review raises the question of whether SAA serves as a biomarker or a causal agent in these diseases.

Sharma et al. conducted a preclinical study using ApoE-deficient mice to test modulators of nitric oxide (NO) bioavailability in diabetic atherosclerosis. The soluble guanylate cyclase activator BAY 60 and stimulator BAY 41 were compared. BAY 60 proved more effective in reducing aortic plaque burden and urinary albuminuria, a marker of renal function, over a 10- to 20-week treatment course initiated after streptozotocin-induced diabetes. These findings support NO-pathway targeting as a therapeutic approach in diabetic vascular and renal disease.

Singh et al. explored phosphorylation changes in NF-κB signaling in endothelial cells exposed to high glucose and hypoxia. Using a large panel of phosphorylation-specific antibodies, they identified 65 modulated phosphorylation sites in 35 proteins. Bioinformatic analysis highlighted increased phosphorylation in two B-cell–related proteins BLNK1 and Bruton tyrosine kinase (BTK). Inhibition of BTK led to an attenuation of glucose induced phosphorylation and activation of PKC and increased IκBα levels, suggesting previously underappreciated pathways influenced by diabetic and hypoxic stress.

Finally, Dai et al. used bioinformatic modeling to analyze co-expression of hub genes in male and female patients with T2DM and CAD (defined as >50% stenosis). Among 16 hub genes associated with immune cell infiltration, NPEPPS (a cytosolic aminopeptidase) and ABHD17A (a protein export gene) were co-expressed and upregulated in CD8+ T cells and NK cell, two immune populations more prevalent in healthy controls. This suggests a potential role for immune modulation in the pathogenesis and treatment of both diabetes and CAD.

Together, these ten studies deepen our understanding of the multifactorial pathways linking diabetes and cardiovascular disease and emphasize the value of diverse research methodologies—from molecular biology and bioinformatics to epidemiology and clinical trials—in unraveling this complex relationship.

